# Decreased expression of BRAF-activated long non-coding RNA is associated with the proliferation of clear cell renal cell carcinoma

**DOI:** 10.1186/s12894-018-0395-7

**Published:** 2018-09-10

**Authors:** Sheng Xue, Sheng-Qun Jiang, Qing-wen Li, Sheng Wang, Jian Li, Shuai Yang, Hai-Min Zhang, Yun-Fei Xu, Long-Sheng Wang, Jun-Hua Zheng

**Affiliations:** 1 Department of Urology, The First Affliated Hospital of Bengbu Medical College Bengbu, Bengbu, Anhui China; 2 Department of Ophthalmology, The First Affliated Hospital of Bengbu Medical College Bengbu, Bengbu, Anhui China; 3 Department of Urology, Shanghai Tenth People’s Hospital, Tongji University, Shanghai, China; 4 Department of Urology, Shanghai General Hospital, Shanghai Jiao Tong University School of Medicine, Shanghai, 200072 China

**Keywords:** Clear cell renal cell carcinoma, Long non-coding RNAs, BANCR

## Abstract

**Background:**

BRAF-activated long non-coding RNA (BANCR) has been associated with various types of cancer. Nevertheless, the role of BANCR in clear cell renal cell carcinoma (ccRCC) is still not fully understood. This study aims to investigate the relationship between ccRCC and BANCR.

**Methods:**

Expression of BANCR in TCGA renal cancer data sets was analyzed. The expression pattern of BANCR in Immortalized normal human proximal tubule epithelial cell line HK-2 and ccRCC cell lines (ACHN, CAKI-1, A498 and 786-O) was detected by real-time quantitative RT-PCR (qRT-PCR). ccRCC tissues with adjacent normal renal tissues diagnosed by pathological methods from 62 patients were used to detect the expression of BANCR, and its correlation with prognosis of ccRCC patients was assessed by Kaplan-Meier method. The LV-BANCR vector was used to examine the influence of BANCR on the proliferation, migration, invasion, apoptosis and cell cycle distribution of ccRCC cells in vitro.

**Results:**

BANCR was downregulated in renal cancer according to TCGA data sets. Compared with adjacent normal renal tissues and normal human proximal tubule epithelial cell line HK-2, BANCR expression was significantly decreased in ccRCC tissues and ccRCC cell lines, and its low expression was associated with poor prognosis. Moreover, in the condition of BANCR overexpression by LV-BANCR vector, the proliferation, migration, invasion capacity of ccRCC cells was inhibited, while the apoptosis was increased and the G1 cell cycle arrest was induced in vitro.

**Conclusions:**

BANCR is downregulated in ccRCC tissues and cell lines, and is associated with ccRCC progression. Thus, BANCR may represent a novel prognostic biomarker and a potential therapeutic target for ccRCC patients.

## Background

Renal cell carcinoma (RCC) is one of the most commonly diagnosed urological cancers in the world [1]. In 2016 in the United States, there were approximately 62,700 cases of renal cancer and renal pelvis cancer that resulted in 14,240 deaths [2]. Among all subtypes of RCC, clear cell renal cell carcinoma (ccRCC) accounts for about 70% of RCC cases [3]. For localized RCC, it has been estimated that more than 25% patients encounter metastases at first visit, while another 25% experience local advancement [4]. Moreover, RCC has shown strong resistance to radiotherapy and chemotherapy [5, 6]. Over the last decade, great progress has been made in genetic and epigenetic variations concerning RCC; Yet, the precise mechanism of RCC pathogenesis still remains unclear. Thus, elucidating the pathogenesis of RCC and identifying available biomarker is of utmost importance.

LncRNAs are no-coding RNAs that are longer than 200 nucleotides, and are characterized by not translated into proteins [7, 8]. Multitudinous long noncoding RNAs (lncRNA) have shown to be involved in various types of tumorigenesis [9–11]. During the last decade, accumulated evidence has demonstrated that lncRNAs have an important role in tumorigenesis, invasion and metastasis of human cancers [12–14]. BRAF- activated non-coding RNA (BANCR) has been originally identified in melanoma cells [15]. BANCR has abnormal expression in various cancers, such as bladder cancer [16], colorectal cancer [17], melanoma [18], endometrial cancer [19], gastric cancer [20], and hepatocellular carcinoma [21]. For example, Wang et al. have reported that BANCR promotes endometrial cancer cell invasion and proliferation by modulating ERK/MAPK signaling pathway [19].

In this current study, we aimed to explore the expression and clinical significance of lncRNA BANCR in ccRCC cell lines and 62 clinical ccRCC samples, and investigate the biological functions of BANCR in ccRCC cells.

## Methods

### Patients and specimens

A total of 62 pathologically diagnosed ccRCC tissues and matched adjacent normal tissue specimens were collected from the Department of Urology, Shanghai Tenth People’s Hospital of Tongji University between 2007 and 2010. The fresh ccRCC tissues and paired adjacent normal tissues were collected from patients underwent radical nephrectomy and were frozen in liquid nitrogen to protect the protein or RNA away from degradation. All diagnosis was pathologically confirmed of clear cell renal cell carcinoma, other pathological patterns of renal cancer were not included in the present study. Patients who have received chemotherapy or radiotherapy before surgery were excluded. The median follow-up time was 34 months until December 2011 and the follow-up was carried out mainly through telephone and outpatient service. The study was submitted and approved by the Ethics Committees of Shanghai Tenth People’s Hospital. Each patient included in the study provided written consent after receiving oral and written information regarding the course and purpose of the study; and all the consents were saved by the ethics committee.

### Cell lines and plasmid transfection

Immortalized normal human proximal tubule epithelial cell line HK-2 was obtained from the American Type Culture Collection (ATCC, USA). Human renal cancer cell lines A498 and 786-O were purchased from the Cell Bank of Type Culture Collection of Chinese Academy of Sciences (CCCAS, China). HK-2 cells were incubated in KSFM medium (Gibco), while RCC cell lines A498 and 786-O were cultured in RPMI-1640 medium (HyClone). Both cell medium were supplemented with 10% fetal bovine serum (Gibco), and all cells were cultured in a humidified atmosphere containing 5% CO^2^ at 37 °C.

Recombinant lentiviruses carrying BANCR sequences (LV-BANCR) and a negative control sequence (LV-NC) were constructed (Hanyin, Shanghai, China). The LV-BANCR or LV-NC was infected into A498 and 786-O cells according to the manufacturer’s protocols. qRT-PCR was performed to detected the expression of BANCR.

### RNA extraction and qRT-PCR

Total RNA from tissues or cells were extracted by using Trizol reagent (Invitrogen, CA, USA) according to the manufacturer’s protocols. For qPCR assay, qualified total RNA was reverse-transcribed to cDNA by SuperScript Frist-Strand cDNA System (Invitrogen CA, USA). The expression of BANCR was measured by real-time PCR using SYBR EX TAQ (Takara). GAPDH was used as an internal control. The prime sequences were as follows: GAPDH, 5’-GTAAGACCCCTGGACCACCA-3′ (forward), 5’-CAAGGGGTCTACATGGCA ACT-3′ (reverse); BANCR, 5’-ACAGGACTCCATGGCAAACG-3′ (forward), 5’-ATGAAGAAAGCCTGGTGCAGT-3′.

### Cell proliferation and colony formation assay

Cell Counting Kit-8 (CCK8) assay was performed to evaluate the proliferation capacity of ccRCC cells. Briefly, A498 and 786-O cells were transfected with LV-BANCR or LV-NC for 48 h, and then seeded into 96-well plates at a density of 5 × 10^3^ per well. After incubation for different time (0 h, 24 h. 48 h, 72 h, and 96 h), 10 ul CCK-8 reagent was added to each well and further incubated for 4 h. At the end, cell proliferation ability was examined by measuring the absorbance at 450 nm, using an enzyme-labeled analyzer.

For colony formation assays, cells infected with LV-BANCR or LV-NC were plated in 6-well plates. 10 days later, methanol-fixed and then stained with 0.1% crystal violet.

### Protein extraction and western blot assay

Total protein was extracted using RIPA lysis buffer added by protease inhibitors and phosphatase inhibitors. After measuring concentration using Bio-Rad assay system, protein was separated by SDS-PAGE minigel and transferred onto Nitrocellulose (NC) membrane (Bio-Rad). The blots were probed with primary antibodies (abcam) overnight and then incubated with secondary antibodies at room temperature for 1 h, signals were detected.

### Flow cytometry

For cell cycle distribution analysis, cells were harvested and fixed in 70% of pre-cooling ethanol overnight. Cells were then centrifuged and resuspended in PBS containing propidium iodide (PI; BD Biosciences) and RNase (100 μg/ml) as well as Triton X-100 (0.2%) for 30 min. Finally, flow cytometry (FACS, BD Biosciences) was used to analyzed cell cycle distribution.

Cell apoptosis were examined using Annexin V-FITC detection kit (BD Biosciences) in accordance with the manufacture’ s protocol. Cells were harvested, washed with PBS and then resuspended with Annexin V-FITC and propidium iodide (PI) stained at room temperature in the dark for 15 min. Flow cytometry was performed to analyzed cell apoptosis rate.

### Wound-healing assay

To determine cell migration, A498 and 786O cells were transfected with negative control or LV-BANCR were seeded into 6-well plates. After the cell reached about 90% confluence, three separate wounds were scratched by a sterile 100 μl pipet tip through the cells. Floating cells were removed by PBS. 24 h later, images of the scratches were got by an inverted microscope (Olympus).

### Matrigel invasion assay

Matrigel-coated invasion chambers with a pore size of 8 μm (Costar, NY, USA) were used according to manufacturer’s direction. After being starved for 24 h, transfected cells were collected. Equal numbers of the indicated cells (5 × 10^4^) were seeded into the upper chambers in 200 μl serum-free medium, while a 600ul medium containing 10% fetal bovine serum was added to the bottom chamber. At 24 h post- incubation, cells remaining on the surface of the upper membrane were scraped off using a cotton swab, and the invasive cells were stained using 0.1% crystal violet for 30 min, and then photographed using a light microscope.

### Statistical analysis

SPSS version 23.0 software (IBM) was used for statistical analysis of the present study. Date from three independent experiments are shown as mean ± SD. The differences between each group were analyzed by a Wilcoxon signed-rank test, a Student’s t test, or a chi-square test. Kaplan-Meier method and univariate analysis was used to estimate the overall survival. *P* < 0.05 was considered statistically significant.

## Results

### BANCR is down-regulated in ccRCC tissues and cell lines

**Fig. 1:**
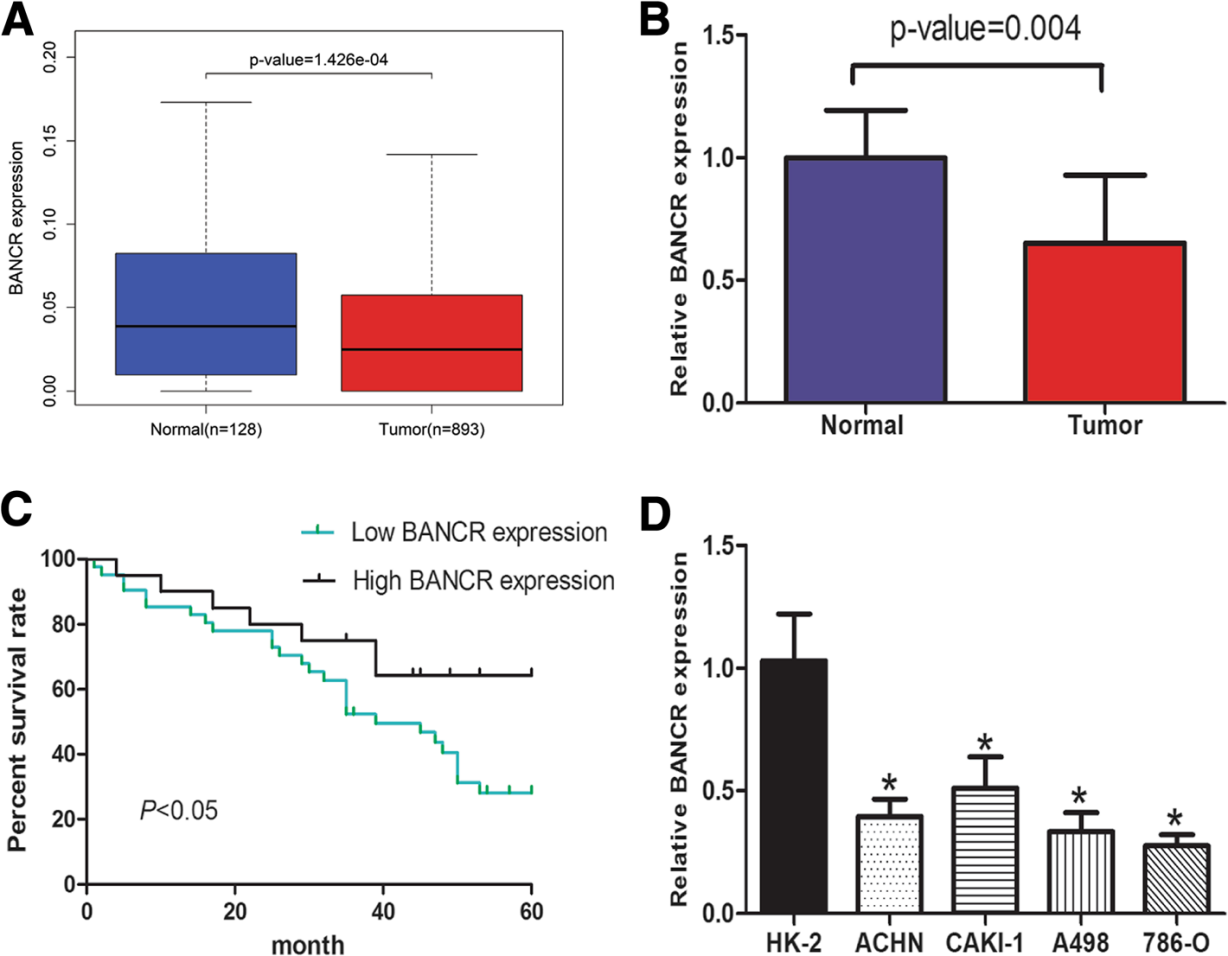
Expression of BANCR in TCGA, ccRCC tissues and cell lines. **a** BANCR expression was lower in renal cancer tissues compared to normal renal tissues from TCGA dataset (*p < 0.05). **b** BANCR expression was lower in clear cell renal cancer tissues compared with adjacent normal renal tissues (*p < 0.05). **c** BANCR expression was associated with overall survival of ccRCC patients (*p < 0.05). **d** High expression of BANCR was found in HK2 cell (*p < 0.05)

We analyzed the expression of BANCR in TCGA renal cancer data sets. The results indicated that BANCR expression was markedly down-regulated in renal cancer tissues compared to normal renal tissues (*p* < 0.01) (Fig. 1a). Furthermore, we examined the expression of BANCR in 62 ccRCC tissues and matched normal renal tissues by qRT-PCR. Briefly, the expression of BANCR was obviously down-regulated in 67.7% (42 of 62) cancer tissues compared with adjacent normal renal tissues of ccRCC patients (Fig. 1b, *p* < 0.01).

To evaluate its clinical significance, we further performed the Kaplan-Meier analysis to evaluate the prognostic significance of BANCR. Our results indicated that patients with higher expression of BANCR had a significant better overall survival compared with patients with lower BANCR expression (log-rank test, *p* < 0.05) (Fig. 1c).

Moreover, we investigated the expression of BANCR in different ccRCC cell lines in vitro. The BANCR expression was significantly decreased in 786-O, A498, ACHN, CAKI-1 cells compared to HK2 cells (Fig. 1d). To sum up, these results revealed that BANCR may suppress ccRCC progression, thus might be a good diagnostic biomarker for ccRCC.

### Overexpression of BANCR inhibited ccRCC cell proliferation in vitro

**Fig. 2:**
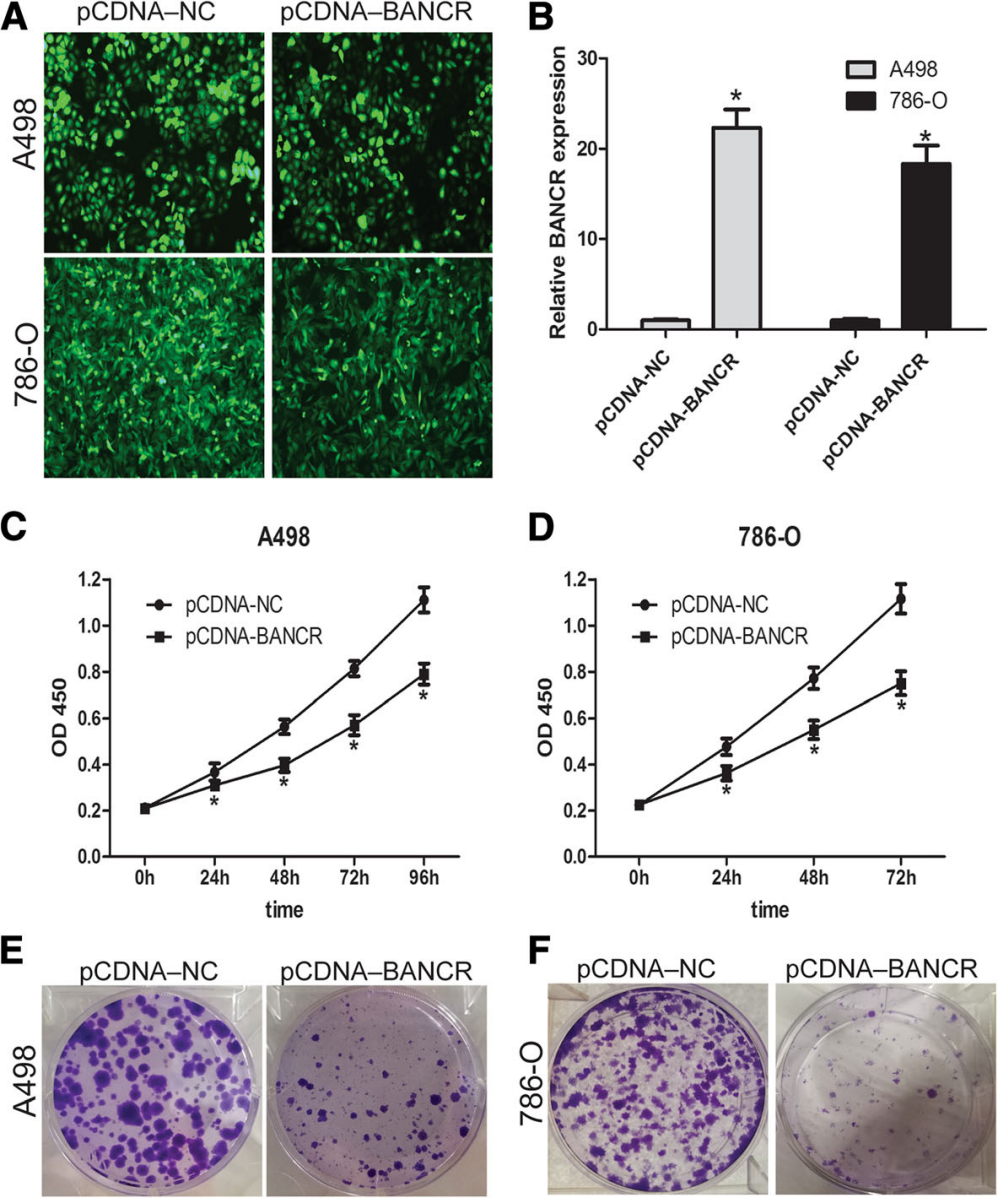
Overexpression of BANCR inhibited ccRCC cell proliferation in vitro. **a** LV-BANCR was effectively transfected into A498 and 786-O cells. **b** The expression of BANCR was examined by q-PCR. CCK-8 assay **(c, d)** and cell clone formation assay **(e, f)** were performed to detect cell proliferation ability of A498 and 786-O cells**.** Data are represented as mean ± SD. *P < 0.05

To explore the possible biological significances of BANCR on ccRCC cells in vitro, we constructed LV-BANCR (cells overexpressing BANCR) and a negative control (LV-NC) cells (Fig. 2a and b). Furthermore, the cell proliferation capacity of A498 and 786-O were detected by CCK-8 assay as well as cell clone assay. Briefly, cell proliferation of 786-O and A498 cells was significantly inhibited by LV-BANCR compared to negative control (*p* < 0.05, Fig. 2c and d). The above results indicated that BANCR may inhibit ccRCC cells proliferation.

### Overexpression of BANCR induced apoptosis and cell cycle arrest in ccRCC cell lines

**Fig. 3:**
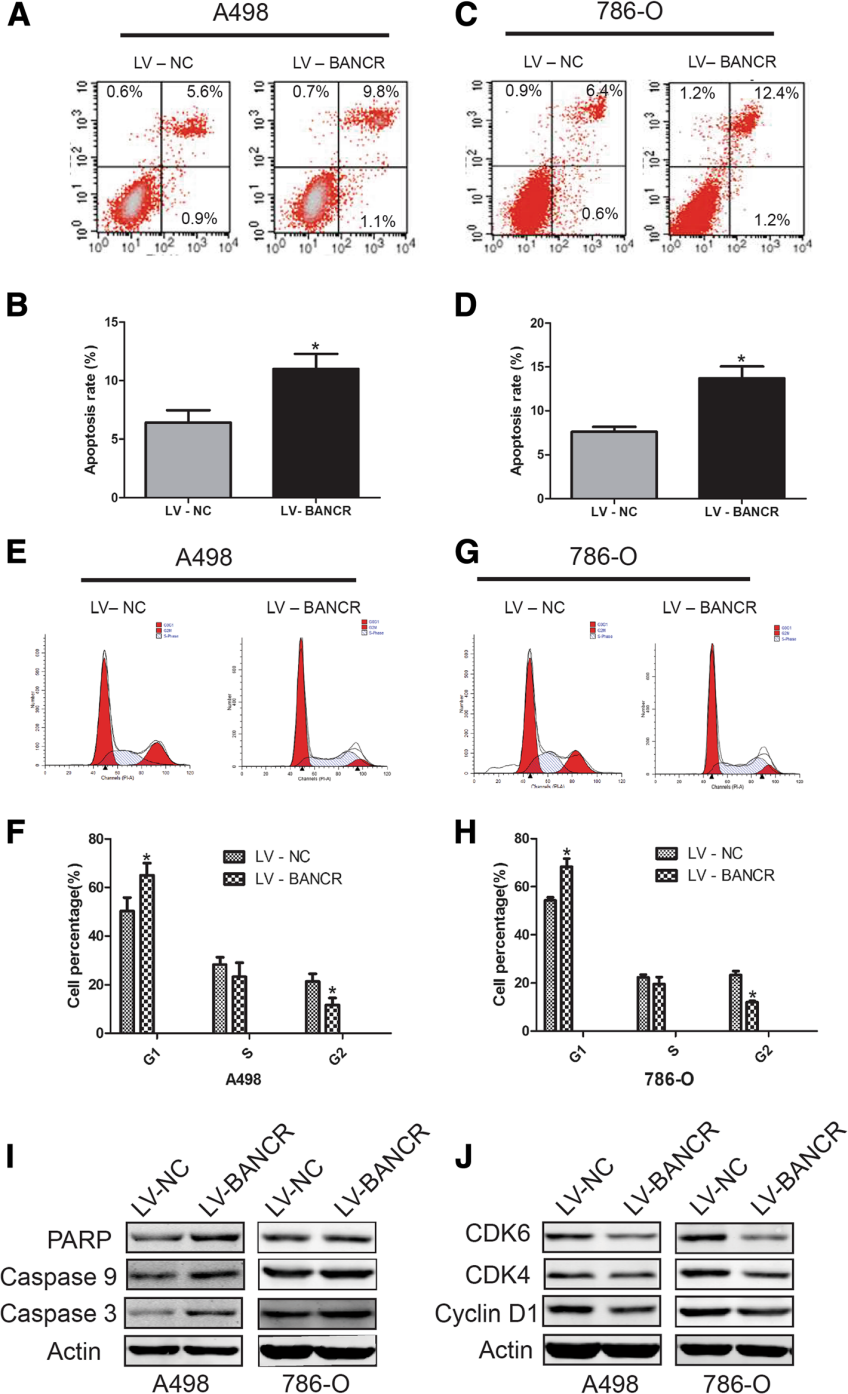
BANCR overexpression induce apoptosis and cell cycle arrest in A498 and 786-O cells. Cell apoptosis of A498 (**a** and **b**) and 786-O (**c** and **d**) after BANCR overexpression was detected by flow cytometry. Cell cycle distribution of A498 (**e** and **f**) and 786-O (**g** and **h**) following BANCR overexpression was determined by flow cytometry. **i** The expression of PARP, caspase 3 and caspase 9 were examined by Western bloting assay in the two cells after BANCR overexpression. **j** The expression of Cycling D1, CDK4 and CDK6 were detected by Western bloting both in A498 and 786-O cells after BANCR overexpression. Data are shown as mean ± SD. *p < 0.05

We further investigated whether LV-BANCR promoted cell apoptosis and/or induced cell cycle distribution. After transfection with LV-BANCR or negative control for 48 h, cells were analyzed by flow cytometry. As shown in Fig. 3a-d, higher apoptotic rate was observed in A498 and 786-O cells transfected with LV-BANCR compared with the LV-NC group. It is well known that members of caspases, including caspase 3 and caspase 9, have a vital role in the process of apoptosis. The cleavage of PARP (nuclear DNA repair enzyme poly (ADP-ribose) polymerase) acts as a biomarker for cell apoptosis, and it is a target of caspase 3. In this study, the expressions of these three proteins were examined in LV-BANCR or LV-NC cells. Briefly, we found that the expression of caspase-3, caspase-9 and PARP were obviously upregulated by LV-BANCR group in both A498 and 786-O cells (Fig. 3i).

Moreover, G0/G1 phase cell cycle arrest was induced in LV-BANCR group in both two cell lines (Fig. 3e-h). Cyclin D1 was closely involved in cell cycle distribution. Meanwhile, CDK4 and CDK6 have shown to play vital role in cell cycle G1 phase [22], thus we further examined their expression in fLV-BANCR or LV-NC cells. It was found that the expression of cyclin D1, CDK4, as well as CDK6 were all downregulate after BANCR overexpression (Fig. 3j).

To conclude, these results suggest that BANCR overexpression causes cell proliferation inhibition by increasing cell apoptosis rate and inducing cell cycle arrest.

### BANCR overexpression inhibited ccRCC cell migration and invasion

**Fig. 4:**
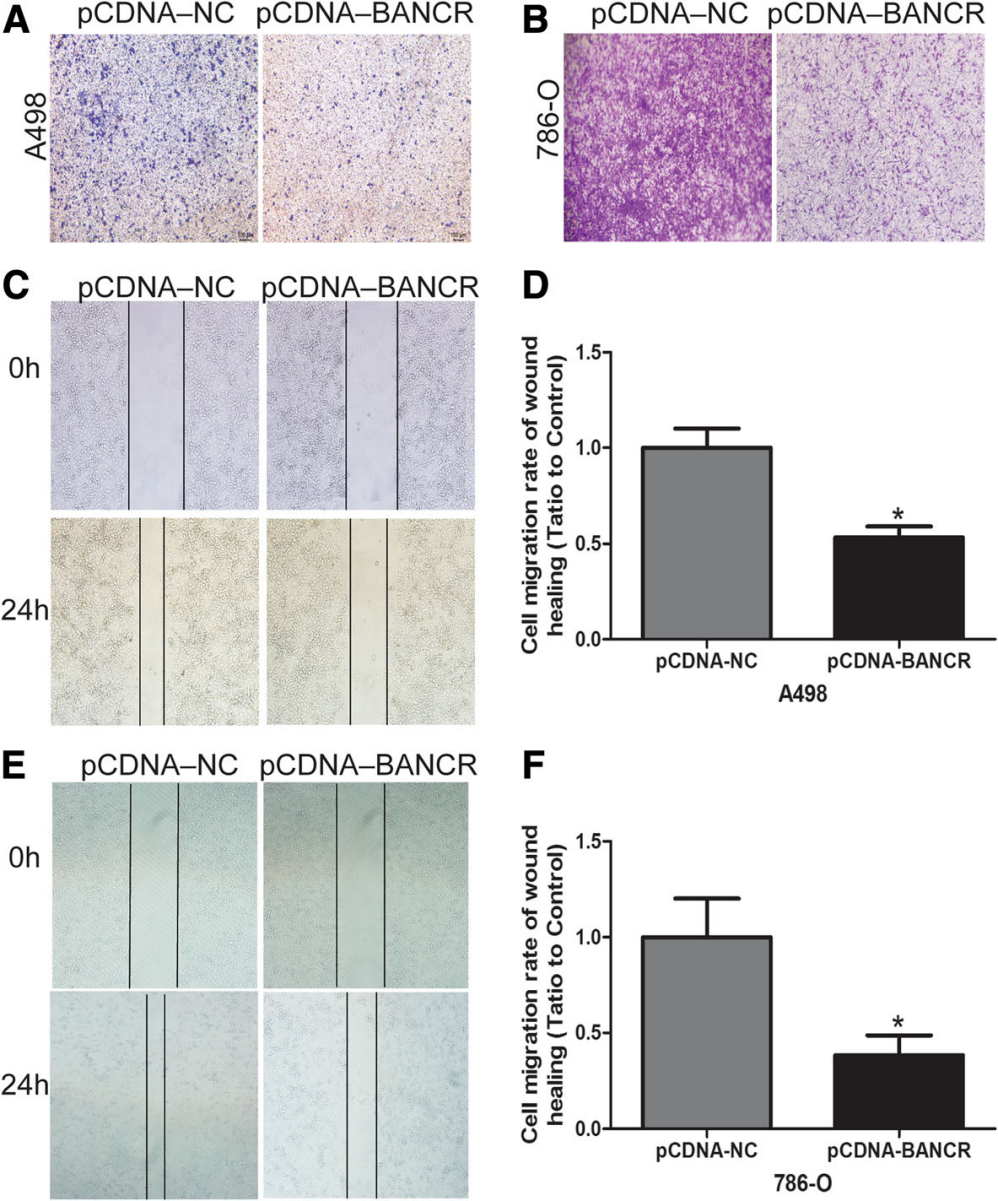
BANCR overexpression inhibited ccRCC cell migration and invasion. The migration ability of A498 (**a** and **b**) and 786-O (**c** and **d**) cell examined using the Wound-healing assay. The invasion ability of A498 (**e**) and 786-O (**f**) cell examined using the Matrigel invasion assay. Data are shown as mean ± SD. *p < 0.05

We explored the effect of lncRNA BANCR on ccRCC cells migration and invasion ability. As for wound healing assay, the migration viability of both A498 and 786-O cells was obviously decreased after LV-BANCR transfection compared with negative control (*p* < 0.05; Fig. 4a-b). Furthermore, BANCR overexpression reduced cell invasion in both A498 and 786-O cells (Fig. 4c). The results indicated that the overexpression of BANCR significantly suppresses ccRCC cells migration and invasion.

## Discussion

Over the last decade, lncRNAs have gained increasing attention as a class of non-coding genes. It has been reported that lncRNAs are essential in tumorigenesis and tumor progression [23, 24]. For example, it has been reported that a higher lncRNA UCA1 expression was found in esophageal squamous cell carcinoma tissues compared with normal tissues, while down-regulation of UCA1 decreases esophageal squamous cell ability of migration and proliferation [25].

As reported, morbidity of RCC is increasing annually [26]. Consequently, early diagnosis based on full understanding of molecular pathways is of great importance for RCC patients. Over the past few years, much attention has been given to the role of lncRNAs in RCC. For example, lncRNA CADM1-AS1 has been reported to be decreased in tumor tissues of ccRCC patients, while a worse survival in ccRCC patients was correlated with relative lower CADM1-AS1 expression [27].

lncRNA BANCR has been reported in various cancers, however, its biological functions as well as prognosis value in different cancer patients were contradictory [28–31]. As a tumor suppressor, BANCR was reported in lung cancer [32, 33]. On the contrary, BANCR was reported to promote tumor growth in various cancers [15, 18, 21, 34]. In contrast, BANCR was shown to be decreased in bladder cancer and colorectal cancer [16].

This is the first study to determinate expression of BANCR and its biological function in ccRCC. Present study results revealed that that BANCR was obviously down-regulated in ccRCC tissues and cell lines. Moreover, the OS (overall survival) was worse in ccRCC patients with low BANCR expression. We further studied the effects of BANCR on the proliferation, migration, invasion, apoptosis and cell cycle distribution of ccRCC cells in vitro*.*

As the weakness of this study, molecular mechanism through which BANCR was decreased in ccRCC and the molecular mechanism involved in BANCR overexpression induced proliferation inhibition in ccRCC cell lines should be further investigated.

## Conclusions

In conclusion, all the above reported evidences suggest that lncRNA BANCR may be a suppressor gene and a novel target gene for the prognosis as well as therapy of ccRCC.

## Abbreviations

ATCC: American Type Culture Collection
BANCR: BRAF-activated long non-coding RNA
CCK8: Cell Counting Kit-8
ccRCC: clear cell renal cell carcinoma
NC: Nitrocellulose
PI: Propidium iodide
